# Clinical providers’ experiences with returning results from genomic sequencing: an interview study

**DOI:** 10.1186/s12920-018-0360-z

**Published:** 2018-05-08

**Authors:** Julia Wynn, Katie Lewis, Laura M. Amendola, Barbara A. Bernhardt, Sawona Biswas, Manasi Joshi, Carmit McMullen, Sarah Scollon

**Affiliations:** 1 Department of Pediatrics, Columbia University Medical Center, New York, NY USA; 2 Medical Genomics and Metabolic Genetics Branch, National Human Genome Research Institute, National Institutes of Health, Bethesda, MD USA; 3 Division of Medical Genetics, Department of Medicine, University of Washington, Seattle, WA USA; 4 Division of Translational Medicine and Human Genetics, Department of Medicine, Perelman School of Medicine, University of Pennsylvania, Philadelphia, PA USA; 5 Department of Pediatrics, The Children’s Hospital of Philadelphia, Philadelphia, PA USA; 6 The Center for Health Research, Rice University, Houston, TX USA; 7 Center for Health Research - Kaiser Permanente Northwest, Portland, OR USA; 8 Department of Pediatrics, Baylor College of Medicine, 1102 Bates St. FC 1200, Houston, TX 77030 USA

**Keywords:** Genomic sequencing, Exome sequencing, Genomic results, Genetic counseling, Secondary results

## Abstract

**Background:**

Current medical practice includes the application of genomic sequencing (GS) in clinical and research settings. Despite expanded use of this technology, the process of disclosure of genomic results to patients and research participants has not been thoroughly examined and there are no established best practices.

**Methods:**

We conducted semi-structured interviews with 21 genetic and non-genetic clinicians returning results of GS as part of the NIH funded Clinical Sequencing Exploratory Research (CSER) Consortium projects. Interviews focused on the logistics of sessions, participant/patient reactions and factors influencing them, how the sessions changed with experience, and resources and training recommended to return genomic results.

**Results:**

The length of preparation and disclosure sessions varied depending on the type and number of results and their implications. Internal and external databases, online resources and result review meetings were used to prepare. Respondents reported that participants’ reactions were variable and ranged from enthusiasm and relief to confusion and disappointment. Factors influencing reactions were types of results, expectations and health status. A recurrent challenge was managing inflated expectations about GS. Other challenges included returning multiple, unanticipated and/or uncertain results and navigating a rare diagnosis. Methods to address these challenges included traditional genetic counseling techniques and modifying practice over time in order to provide anticipatory guidance and modulate expectations. Respondents made recommendations to improve access to genomic resources and genetic referrals to prepare future providers as the uptake of GS increases in both genetic and non-genetic settings.

**Conclusions:**

These findings indicate that returning genomic results is similar to return of results in traditional genetic testing but is magnified by the additional complexity and potential uncertainty of the results. Managing patient expectations, initially identified in studies of informed consent, remains an ongoing challenge and highlights the need to address this issue throughout the testing process. The results of this study will help to guide future providers in the disclosure of genomic results and highlight educational needs and resources necessary to prepare providers. Future research on the patient experience, understanding and follow-up of recommendations is needed to more fully understand the disclosure process.

**Electronic supplementary material:**

The online version of this article (10.1186/s12920-018-0360-z) contains supplementary material, which is available to authorized users.

## Background

Genomic sequencing (GS) is established as an effective tool for clinical diagnosis, research discovery and, increasingly, precision medicine [1–4]. Tens of thousands of people have had diagnostic and/or research GS and in the coming years over one million people will have GS through studies such as All of Us and the Clinical Sequencing Evidence Generating Research Consortium [5, 6]. While great strides have been made, research on how to responsibly integrate genomic medicine into clinical care, particularly the practice of disclosing these results to patients and research participants, is still evolving.

The Clinical Sequencing Exploratory Research Consortium (CSER) [6] comprises a group of NIH-funded projects that was formed to explore the key challenges of integrating GS into clinical care including: 1) the generation, analysis and interpretation of GS data; 2) the translation of these data to clinical care; and 3) the ethical, legal and social implications of genomic medicine. The CSER consortium Genetic Counseling Working Group (GC WG) was formed in 2012 and is comprised of counselors with experience consenting and returning results to over 5000 research participants and patients [7]. The CSER GC WG is focused on addressing the challenges encountered by GCs in genomic medicine including consent, genomic education, results disclosure and the psychosocial needs of the participants.

Provider and patient perspectives on the consent process for GS studies have been described by the CSER GC WG and others [8–11]. These studies have identified key elements of informed consent including: scope and process of the study, genomic education, physical and mental health benefits and disadvantages, family impact, confidentiality and security of data, and secondary findings [12]. Challenges of the informed consent process have also been reported on, including navigating patient expectations of the expansive nature and diagnostic potential of GS and the possibility for uncertainty both with diagnostic results and secondary findings [13, 14]. Clinicians obtaining informed consent for GS report spending a portion of the session fostering realistic expectations and correcting potential misconceptions about the current state of genomic knowledge - topics that are not typically covered in consent for traditional genetic testing [11, 15].

As best practices for informed consent for GS are being finalized, evaluation of the return of results (RoR) process has begun. The CSER GC WG previously published a case series that illustrated anecdotal challenges unique to disclosing GS results including returning large amounts of information, returning uncertain results, and disclosing predictive secondary results without the context of an existing diagnosis [13]. Typical challenges of traditional genetic testing were magnified by the expansive nature of GS such as navigating the atypical presentation of known conditions. The phenomenon of the “nuanced negative”, which refers to the notion that a negative result from GS is dynamic rather than finite, is another unique characteristic of genomic results [16].

We sought to expand upon the themes described previously and explore novel ones in this study by conducting semi-structured interviews with healthcare providers who are returning GS results from CSER projects. The interviews provided the opportunity to gain insights into RoR sessions, including how providers prepare, common patient reactions, and how experience with this process has changed providers’ practices. We also explored training needs that will help inform the preparation of a larger workforce to manage the predicted increase in the volume of these results. These results will enhance the developing guidelines for RoR for GS and will help to educate the broader health care community as genomic medicine is integrated into health care.

## Methods

### Recruitment and participants

Representatives of 11 CSER sites (www.cser-consortium.org) (10 U awards and 1 R award where results were returned to participants) were asked to identify study providers returning GS results to participants. Thirty providers, encompassing a representative sample of the provider types including genetic counselors, geneticists, non-geneticist physicians and nurse practitioners returning results at each site, were contacted by the study investigators up to three times via an email which briefly explained the study and invited them to participate. If there was no response following 3 attempts, the provider was assumed to be a passive decliner.

The study was approved by the Baylor College of Medicine Institutional Review Board.

### Methods

Providers were first asked to complete a brief survey with questions about their professional background, years of experience, the number of results they had returned and details regarding their CSER site participant population and RoR policies (Additional file 1). An introductory letter explained that receipt of the returned survey to the study PI served as consent by the respondent to be contacted by study staff to schedule a telephone interview.

Each respondent then completed a semi-structured telephone interview about their experiences returning results. The interviews were conducted by one of six trained study investigators (LA, BB, SB, JW, CM, MJ) who used an interview guide to facilitate the conversation. Verbal consent was obtained at the start of the interview. The interview guide consisted of open-ended questions and prompts about preparing to return results, RoR session structure and contents, participant reactions to sessions, follow-up processes, and training needs (Additional file 1). To ensure reliability across the interviews, the audio recording of the first interview completed by one investigator (MJ) was reviewed by the other interviewers and a training conference call was held to discuss interview technique and review the interview guide in detail. Four additional pilot interviews were then completed and a second conference call was held to address areas of difficulty within the interview guide. Only minor changes to the script were made before proceeding with additional interviews. The interviews were audio-recorded, transcribed and de-identified. All interviews took place between May 2016 and September 2016 and lasted between 23 and 90 min.

Interview transcripts were coded using QSR international’s NVivo 11 software [17]. A draft codebook was developed by one investigator (KLL) based on a review of several transcripts, the interview guide, and research objectives. Three study investigators (MJ, KLL, SS) used the draft codebook to code four completed study transcripts and iteratively reviewed and revised it as described by Braun et al. [18]. The three investigators coded and reconciled any discrepancies in an additional four transcripts. The remaining transcripts were coded by only one investigator (either MJ, KLL, or SS). Upon completion of the coding, the data were analyzed for common themes and relationships between them, and exemplary quotes were identified.

## Results

### Respondents

Thirty providers from 9 CSER U award projects and 1 R award project were approached for participation and 21 consented to the study and completed the survey and interview (Table 1) for a 70% response rate. The study cohort included 12 genetic counselors and 9 other healthcare providers (physicians and a nurse practitioner) 4 of whom were medical geneticists (Table 1). Those providers who actively or passively declined included 3 genetic counselors, 2 geneticists and 3 other healthcare providers. Most (81%) of the providers had been practicing for over 5 years, and more than half had been involved in greater than 50 results disclosure sessions at the time of the interview (Table 1). Two or three providers from each project were interviewed with the exception of three sites where only one person was interviewed.

**Table 1:** Characteristics and experience of the 21 participants

	Number	Percent
Profession		
Genetic Counselor	12	57%
Other Healthcare provider^a^	9	43%
Geneticist	4	19%
Non-Geneticist	5	24%
Years of Experience		
0–5	4	19%
6–10	8	38%
11–15	1	5%
16–20	3	14%
> 20	5	24%
Number of Result Disclosures		
1–50	9	43%
51–100	4	19%
101–150	5	24%
151–200	1	5%
> 200	2	10%

^a^Other healthcare provider includes physicians and a nurse practitioner

The participants in this study are referred to as respondents to differentiate them from the participants in the CSER consortium studies. In CSER projects which include children, participants are sometimes referred to as parents or families by the respondents. Diagnostic results are defined as results related to the clinical indication of the study participant, which varied by CSER site. Secondary results are defined as results unrelated to the clinical indication. Secondary results were the only type of result for healthy participants. The type of secondary results returned varied by study site but included the American College of Medical Genetics and Genomics list of secondary findings [19, 20] and sometimes also included other conditions associated with a risk for a genetic disease. Pharmacogenetic results and carrier results were also returned by some sites [21].

### Logistics of returning results

All results where a genetic variant (diagnostic, secondary, carrier) was identified and most results where no variant was identified were disclosed in person. The type of provider present at disclosure varied by site, and included GC only, GC and another provider or another provider only (Table 2). Respondents, both genetic and non-genetic providers, reported that it takes 10 to 90 min to prepare for a result disclosure session. Respondents agreed that disclosure of unfamiliar results, variants of uncertain significance (VUS) and medically actionable secondary results take longer to prepare for, whereas negative results and positive diagnostic results take less time. In order to prepare for disclosure sessions, respondents commonly used online resources such as OMIM® [22] GeneReviews® [23] or primary literature on the variant(s) or conditions, and roughly half of the respondents also used formal study meetings as part of their preparation. Most respondents met routinely with multidisciplinary study teams to discuss findings prior to RoR sessions, although some only met if the results included a medically actionable result or secondary result. Less frequently, the respondents searched for support groups or local specialists where they could refer their participants.

**Table 2:** Participating Clinical Sequencing Exploratory Research (CSER) sites

CSER Site	Patient Population	Disease	Mode of Delivery
Baylor College of Medicine	Pediatric	Cancer	Oncologist with genetic counselor present for consult as needed
Brigham and Women’s Hospital	Adult	Healthy and cardiomyopathy	Primary-care physician or cardiologist
Columbia University Medical Center	Adult	Healthy/Not disease specific	Geneticist and genetic counselor
Children’s Hospital of Philadelphia	Pediatric	Variety of pediatric diagnoses	Genetic counselor and/or medical geneticist, cardiologist, hematologist, neurologist
Dana-Farber Cancer Institute	Adult	Cancer	oncologist with a referral to genetic counseling if needed
Hudson Alpha	Pediatric	Developmental delay and/or intellectual disabilities	Medical geneticist and genetic counselor
Kaiser Permanente	Adult	Healthy/Preconception carrier testing	Genetic counselor
University of North Carolina	Adult and Pediatric	Variety of diagnoses	Medical geneticist and genetic counselor
University of Washington	Adult	Cancer	Genetic counselor only or genetic counselor and medical geneticist
National Human Genome Research Institute	Adult	Healthy and atherosclerotic heart disease	Genetic counselor and/or medical geneticist

Reported RoR session lengths ranged from 10 to 90 min and were generally longer if the session included: multiple results, diagnostic results, medically actionable secondary results, or more questions from participants. Typically, when results were disclosed by both a physician and genetic counselor, the physician led the discussion of the diagnostic results and the genetic counselor reviewed any secondary results. Less commonly, a genetic counselor was not involved (only one site) or results were disclosed in multiple sessions, typically when there were both diagnostic and secondary findings.

### Content of return of results disclosures

There was diversity across CSER sites in types of results returned based on patient population and study design (Table 3). Most respondents used their site specific laboratory report to guide the session content. Some respondents reported providing information on every condition on the report, while others reported providing a more general overview. The return of no diagnostic results and patient misconceptions of GS technology were the main factors reported to alter the content of sessions. In these sessions, more time was taken to address the limitations of genomic technology.

**Table 3:** Results types returned by site

	Result Types			
CSER Site	Diagnostic	Secondary	Carrier^a^	PGx
Baylor College of Medicine	P, LP, VUS	P, LP	Yes	Yes
Brigham and Women’s Hospital	P, LP, VUS-Favor pathogenic	P, LP, VUS-Favor pathogenic, common complex for cardiometabolic traits	Yes	Yes
Columbia University Medical Center	NA	P, LP	Yes	Yes
Children’s Hospital of Philadelphia	P, LP,VUS	P, LP	Yes	No
Dana-Farber Cancer Institute	P, LP, VUS	P, LP	Yes	No
Hudson Alpha	P, LP, VUS	P, LP	Yes	No
Kaiser Permanente	N/A	P	Yes	No
University of North Carolina	P, LP, VUS	P, LP	Subset of adult participants	Subset of adult participants
University of Washington	P, LP, VUS	P	Yes	Yes
National Human Genome Research Institute	P, LP	P, LP, VUS	Yes	Yes

*Abbreviations*: *CSER* clinical sequencing exploratory research, *P* pathogenic, *LP* likely pathogenic, *VUS* variant of uncertain significance, *PGx* pharmacogenetics, *NA* not applicable

^a^Number of recessive conditions tested for by site varied

Some respondents explicitly discussed the possibility of re-analysis in the disclosure session when a diagnosis was not identified or in the discussion of variant of uncertain significance (VUS) results particularly if they had experienced previous cases where re-classification occurred. Others reported more generally discussing the possibility that we may learn more about GS findings over time. At one site, the results letter had a statement regarding the possibility of variant reclassification but it was not routinely discussed in the session. Few providers had been involved in returning reclassified results at the time of interview.

The most common educational materials used during the session and provided to the patient were visual aids to explain inheritance patterns and condition specific resources. Genetics providers were more likely to report the use of educational materials during the session than non-genetic providers. Respondents reflected on how experiences in earlier sessions during the study period informed improvements to existing materials including simplification of reports and letters as well as development of novel materials. Some examples of educational materials developed during the study included visuals aids explaining the limitations of testing, carrier status handouts, and concise coversheets to reports summarizing key findings in a bulleted format. All sites provided participants with a copy of a laboratory report following the result disclosure and most provided a letter that summarized the result(s).

In general, most sites reported that there were limited follow-up questions from participants subsequent to the visit. If follow-up contact occurred, it was typically related to a change in family health history or a new child being born in a family. In the absence of participant follow-up, some respondents promoted the utility of follow-up calls to check-in on participants following RoR.

### Participants reactions to results

Respondents reported that participant reactions to GS results varied widely, ranging from enthusiasm and relief to confusion and disappointment. Reactions were influenced both by the specific results and the patient’s health status and experiences with medical care. Examples of patient reactions by result type can be found in Table 4.

**Table 4:** Respondents’ reflection on how type of result influences participant reactions to results

Type of result	Importance	Illustrative quote(s)
Positive diagnostic results	End to the genomic odyssey, relief to have an answer, disappointment and worry about the prognosis, frustration or disappointment because of limited information and resources available.	*“I can see them relax in the room…it is a relief to understand what is going on”* (E02) *“[the mother] had become very happy with this sort of slow steady progress her daughter was making…. not knowing kind of meant there was no cap on how far and how well her daughter could do. All of a sudden, getting a diagnosis, it surprised even her that her reaction was kind of, oh crap, is there now going to be a limitation?”* (F02) *“Oftentimes we think we have the answer for a patient’s symptoms, but because the condition is so rare …the answer isn’t wrapped up neatly… so while folks are excited to get an answer, sometimes it’s frustrating because they expected an answer to open things up to new treatment or contacts with parents who have kids who have the same disease or just a wealth of information that they would be able to unlock.”* (F03)
Secondary results	Curiosity, confusion, misinterpretation, information overload when identified. Relief or disappointment when not identified.	*“I would say our most common participant reaction is, ‘Oh that’s really cool’. They really think it’s interesting they’re excited. They say it satisfies their curiosity… our patients they self-select… They want the information. They’re really enthusiastic for it*.”(B01) *“But, returning secondary results is different than a diagnostic result or a result in which someone has a family history of it, which they have context that can apply to the result. In these results, they don’t have any context, unless perhaps they’ve seen someone or met someone with it...”* (J01) *“The incidental findings have been unique in our population because I think they are already dealing with such a crisis that for many of them, I actually worry more about the fact that they’re not going to follow up on the incidental finding in the context of everything else that’s going on.*” (D03)
Uncertain diagnostic results	Confusion about implications for medical care and risk assessment, tolerance of ambiguity.	*“They say, ‘Wait a minute. Regardless of what this is, you say you found this gene. This gene’s causing cardiomyopathy. How can I say this isn’t part of his problem?’ We’re looking at them and we’re like, ‘Yeah, you’re right. We don’t know.’”* (I01) *“I think it’s a tolerance of ambiguity issue more than a trust issue. I think some people have a greater tolerance of ambiguity than others.”* (C02)
No diagnostic results	Disappointment, unmet expectations, confusion, acceptance, or relief of no diagnostic results.	“*Parents who go to internet, who talk about this, read about this, they think that with this we can find a cure, we can find a magic change. I think that’s abuse of this technology, by companies and many physicians as well.”* (D02) *“Because that has really been their experience so far within the genetics community, that often they have felt like an unsolved mystery, so it’s not a surprise to them not to get a complete and clear answer from one test.”* (F03) *“They weren’t psychosocially ready for a diagnosis and I think that they had children who weren’t profoundly delayed, and had some hope that their children maybe didn’t have the issues that they had, that they would grow out of them. They were actually quite happy with a negative result because they weren’t quite ready to accept the challenges in place.”* (E03)

#### Diagnostic results

When returning diagnostic results, respondents recounted that some participants were relieved to finally have an explanation for the diagnosis, especially when the finding was consistent with the clinical presentation or the family history. However, respondents indicated that participant reactions were sometimes more mixed when the results confirmed or raised fears of a condition that is progressive or indicated an unexpected poor prognosis for a child. The diagnosis of a rare condition was particularly challenging because there was limited or no information about clinical management and no immediate access to patient support groups.

#### Secondary results

The return of secondary results also caused variable participant reactions. The most common reported participant reaction was disappointment when no secondary results were identified and, when results were identified, participants were often excited to learn about the result. Respondents attributed these reactions to the participants’ motivations to participate in the study – one of curiosity and information seeking. For other participants, respondents reported that secondary results were surprising and anxiety producing and they sometimes had difficulty conceptualizing the associated risks. Respondents indicated that, when disclosing medically actionable secondary results, they tried to emphasize the benefits of having these results and the ability to take preventative measures. One respondent reflected that participants who are overwhelmed by diagnostic results and/or have ongoing medical issues may have trouble processing the implications of secondary results. Those receiving both diagnostic and secondary results may become disengaged if all results are disclosed in a single session. However, sites were faced with the challenge of balancing the potential for information overload with the practical needs of participants such as juggling multiple medical visits, time off work and the cost of travel and parking.

#### Uncertain results

Several respondents mentioned the unique and layered complexity of VUS identified by GS. They reported that it was a challenge to facilitate participant understanding of the uncertainty of the pathogenicity of a VUS and guide them through the implications of these types of results. Many participants had positive or neutral reactions to VUS results, particularly families who had been on a diagnostic odyssey and had previous experience with testing that did not yield a diagnosis. Though, for some participants, it was difficult to accept that a VUS may not be the cause of their condition. Some over-interpreted the VUS to be relevant to their own health or family history.

#### No diagnostic results

Reported reactions when no diagnostic results were identified ranged from disappointment to relief. Families or participants who had high expectations that GS would find an explanation for a child’s or their own condition were often disappointed when there was no diagnostic finding. Similar to reactions to VUS results, respondents reflected that families on a diagnostic odyssey may have had more modest expectations of the testing and therefore more muted disappointment. On the other hand, respondents reported that some participants and family members were relieved to have no diagnostic findings. For example, the family may interpret the absence of diagnostic findings as support that a diagnosis was unlikely to be genetic and other family members or future children were unlikely to be at risk or that hope remains about their child’s prognosis.

#### Variant reclassification

Although few providers had been involved in returning reclassified variants at the time of interview, those who had reported this seemed to be understood and tolerated by the participants. Respondents had disclosed both variants that had been upgraded to pathogenic as well as those that had been downgraded to benign. Some respondents felt that offering variant reclassification built trust between the participant and provider by demonstrating that their case was being managed with ongoing care. Others discussed scenarios in which variant reclassification may have a potential negative impact, such as when multiple family members had been tested or participants had undergone screening for a condition only to have a variant reclassified from pathogenic to a VUS or benign. However, at the time of interview many respondents discussed these concerns in a hypothetical sense and reflected that understanding these effects would be an important area of study for the future.

#### Expectations of sequencing

Despite the variability of reported reactions, respondents frequently noted that participant reactions were often colored by the unrealistic expectation of getting highly useful information from GS. Some respondents speculated that these unrealistic expectations may be fostered by the portrayal of this technology in the media and the hype that has developed around it. Families or participants who had high expectations that sequencing would find an explanation for a condition or would identify a secondary result were often disappointed when there were no results.

### Lessons learned & Evolution of the process

Many of the challenges to returning GS results related to the number, complexity and uncertainty of results as well as managing unmet expectations of the testing (Table 5). Respondents were asked to reflect on the evolution of the RoR process over the course of the study and how they addressed these challenges. Respondents reported that changes were informed by experiences in informed consent and RoR sessions, familiarity with the types of results and overall experience and knowledge gained through their work on the study. Respondents found that it took less time to prepare as they became more familiar with the types of results and the relevance to the participant. Reduction in preparation time was also influenced by development of additional resources as the study progressed, such as internal databases of variant interpretation, disease descriptions and letter templates. One respondent reflected on how preparation time changed over the course of the study.

*“Certainly that the amount of time it takes to prepare for a result session and to write reports has come down because we have done so many of them. You often have something you can build from, even if you haven't had a variant in that gene before, if you've had a variant in a gene similar and you've got some of that text done and you have some of that experience.”* (E03)

Many respondents reported that the disclosure sessions became less structured and more flexible in both order and content over the course of the study. Some respondents indicated the content of the sessions also became more personalized to the participant as the respondent became more familiar and comfortable with the types of results.

**Table 5:** Respondents’ reflections on challenges of returning genomic results and methods used to address challenges

Challenge	Method to Address Challenge	Illustrative quote
Multiple results	Re-iteration and restating results. Open ended questions to assess understanding. Multiple sessions. Follow up communication.	*“I think probably the biggest challenge is when you have a lot of results on the report, going through those all in one session. I think sometimes there have been sessions where I’ve given back a diagnostic and an incidental finding in one session and frankly, I just feel like that overwhelms the families….The other part is we’ve noticed over time that the families aren’t really engaged in the sessions and they’re not asking a lot of questions.”* (D03)
Unmet expectations	Explore and set realistic expectations in the consent session. Acknowledgment and validation of feelings of disappointment and frustration.	*“We try to tell them, we’re not going to give up the search, and we’re going to remain curious about their child, and I think that winds up helping alleviate some of that tension that they know that someone’s still interested in their child, even though there was a negative result”.* (E02) *“They [patients] may have some unrealistic expectations about what exome sequencing or genome sequencing would be able to tell them. That the future would be predicted and that it may be difficult to ground them in the reality of what we know and what we don’t know.”* (C02)
Uncertainty	Review of current limitations in genomic knowledge. Reassurance that communication pathways are open and updates may be available.	*“Some of these are novel variants that have not been seen before. It’s really hard to help patients understand sometimes that we aren’t 100% sure that if a child were to inherit this gene change along with another gene change in that gene ... First of all, if they would have disease and second of all, where they would be on the spectrum…. I think they have a lot of assumptions about how clear and concrete all of medicine, if not genetics, really should be.”* (C01)
Unanticipated Results	Facilitate feelings of empowerment to have this knowledge. Ability to seek early screening and prevention or plan for the future	*“Navigating that surprise and trying to present it as both good news in a way - that is, ‘we didn’t cause this, and you have this, and it’s really good we now know about it’. But it doesn’t make for a good day, to get an incidental result, even if it in reality is a good thing to know about.”* (F01)
Communication of results with family members	Encourage reflection of this in the consenting session. Make a plan in the disclosure session.	*“We spend a lot time talking about that, trying to anticipate the benefits and the downsides of sharing information with particular family members. Trying to anticipate how that’s going to make my patient feel…..A lot of people, they are information seekers, they want all their family members to be tested and sometimes it’s really hard for them to cope with the fact that their family members may be dismissive of this information. We have to spend time talking about strategies for dealing with that. Understanding why their family members might feel that way” (B01)*
Overwhelmed or not engaged	Anticipate, acknowledge, foster a relationship of ongoing communication and options for follow up conversations.	*“Acknowledging their struggle and giving them space and giving them time and telling them that they don’t have to remember this all today and they don’t have to talk about all of this today, if they don’t want to.”* (A02) *“I’ve listened to these conversations enough, so I do try to anticipate what they might ask and go ahead and put it out there, even if it’s something they might be uncomfortable with*” (E02)
Provider’s expectations	Recognize one’s own biases and misconceptions. Reassess one’s own at regular intervals.	*“... Sometimes the most concerning results to you are not the ones for the patient*.” (J01) *“I was struck by how much patients enjoyed the journey of the genome. They enjoyed understanding over time, rather than having everything ‘Oh here’s your test results. Bye bye.’ The thing that was most important about the genome was engaging the person over time”.* (G03)

Respondents also reflected on the continuum of the consenting and disclosure process and how experience with both sessions informed and led to changes in the other. Respondents reported spending more time with participants in the consenting session to establish realistic expectations, emphasize the limitations of the testing, and prepare them for the potential of uncertain results and the possible need for additional testing. One responded reflected on how the consenting process changed.

*“I also try to prepare them for the uncertainty. We're doing all of this in the context of research so we may return to them things that we don't fully understand. Variants, some fall out from that uncertainty, how do you think you would feel if I told you I'm not sure what your risk for cancer is, I think you're at a higher risk but I don't know. Helping them anticipate that and see if it's really for them.”* (C01)

Respondents also discussed how the disclosure sessions evolved to anticipate and correct misconceptions. Several respondents discussed how some participants over interpreted results, especially when they did not receive diagnostic results. For example, to address the tendency of participants to misinterpret carrier results as incurring a personal risk, one respondent commented on how she reiterated that there were no direct health implications of these types of results.

*“I try to maybe over-explain carrier status because I think when that was the only finding, again, they were getting that confused or thinking their child will someday develop this recessive condition.”* (D01)

Another commonly reported challenge was that participants had difficulty formulating questions in the sessions either because there were no diagnostic findings, the participants were overwhelmed with the results or their current situation, or the participants were not actively engaged in the session. Several respondents addressed this by sharing how other families have responded to results and the types of questions other families have asked as a way to activate involvement in the session when participants became overwhelmed and disengaged.

Respondents also needed to provide guidance in order to prepare the participants for sharing the results with family members. Navigating participants through the process of anticipating the possible reactions of their family members was a reported challenge.

One component of the evolution of respondents as practitioners was reflecting on their own misconceptions. For example, respondents sometimes commented on their expectations when entering into this process and the ways in which those expectations were challenged, such as feeling surprised when patient responses to results contested their own biases. One respondent reflected on this.

“*from one family to the next, you might have frustration, anger, relief, grateful for the opportunity to participate in research, curiosity, all kinds of different reactions for essentially, the same information. That has, and that these participants and our patients continue to surprise me with their ability to challenge my biases, in that regard*.” (E01)

### Similarities to traditional genetic testing

While respondents highlighted the differences of the results disclosure sessions from traditional genetic testing, they frequently also reflected on the similarities, especially when they had more experience with disclosing results.

“*Overall, it is surprising how little difference it makes that we're doing an exome sequence, in that a lot of things that we thought we would have to explain because of this big test such as the methodology, it's just turned out really not to be the case*.” (F03)

Respondents reflected on the benefits of using traditional genetic counseling skills including contracting with the participant to create a mutual agenda for the session, addressing verbal and non-verbal cues, re-iterating and restating information, asking open-ended questions to assess understanding, bringing up frequent misperceptions, acknowledgement and validation of the participants’ emotions and experience, establishing a trusting relationship and providing opportunities for future contact [24].

*“When I do that contracting bit at the very beginning a lot of times I can tell if somebody is uncomfortable waiting even 30 seconds more to hear what the result is. You can just tell by their non-verbal cues how anxious they are…you can tell, this person can't wait any longer in which case we'll just jump right into the results or at least give them enough of a nugget that they can hold on.”* (B1)

As in disclosure sessions for traditional testing, respondents also reflected on the importance of validating participants’ reactions, building a relationship of trust with the participant to engage them and to facilitate learning and helping the participant to understand that the relationship extends beyond the session by offering follow up sessions. This is particularly important when there are multiple results, uncertain results or no diagnostic results in the setting of a suspected genetic condition.

### Training needs

Respondents were asked to reflect on their experiences and how future providers – both genetic and non-genetic – can prepare to return genomic results. Several respondents expressed some hesitation about whether or not providers, particularly non-genetic providers, currently have the necessary training and resources to incorporate GS into practice. Particularly, they were concerned about education on appropriate indications for GS as well as the limitations of the technology and how this impacts the interpretation of a negative genomic analysis. Some felt that the newer generation of providers might be better prepared. Though, even with adequate education, there may not be sufficient time allotted to non-genetic providers to return results for GS. Several respondents felt that the ability to triage the amount of information in a GS report relevant to a patient, such as avoiding unnecessarily detailed discussion of VUS or carrier results for a patient not presently considering family planning, would be an important skill to learn. Despite concerns, several respondents acknowledge a role for non-geneticists especially because genetic providers are a limited resource.

“*I think there's definitely a role for them* [non-genetic providers]*. I think that ... I mean, I think we're going to have to give up some ownership of some of our roles with our patients because there just aren't enough of us to do all of this. I think some of the more straightforward cases or maybe some of the more straightforward pieces of cases and things that you could quickly and easily educate non-genetics providers about and confirm their competency about, are things that they could be informed about.”* (C01)

Several expressed caution in creating genetic exceptionalism and felt that in some cases a non-genetics specialist returning results relevant to their specialty, such as a cardiologist returning cardiac results, might be just as effective as a geneticist. The non-genetics respondents did express increased comfort with the disclosure process over time as they became more familiar with the reports and types of results being returned. One non-genetics respondent reflected on how his/her understanding improved over the course of the study.

“*In the beginning because there are different categories of mutations, changes, and I think in the beginning the definition of those different categories were not so clear to me. After some experience, they were much clearer*.” (D02)

Genetic and non-genetic respondents alike emphasized the importance of improving awareness and availably of additional resources including provider-to-provider consultation and electronic resources. Non-genetics providers did reflect on the increased confidence that came with having access to a study team including genetics experts, expressing some concern for those non-genetics providers without such resources. The availability of a referral to a genetics professional, for non-genetic providers who have limited comfort with some types of results and time constraints to discuss them with the patient, was especially important.

“*I think it's going to be important for our non-genetics professionals to know where they can go to ask questions and have a resource that they can kind of phone a friend essentially when they get results back and there's something that they're unfamiliar with.”* (E03)

## Discussion

Through interviews with clinicians with considerable experience returning results from GS as a part of the CSER consortium, we identified common practices utilized by these first adopters primarily positioned in large academic medical centers. The variability in patient reactions to results and the factors affecting these reactions as well as challenges that should be addressed as GS moves increasingly into clinical care were described by respondents. Some of the challenges will be minimized as clinicians gain more experience returning results from GS, variant databases improve and educational materials and counseling aids are developed to address patient expectations and educational needs. Finally, as more clinicians begin using GS, experienced providers, including those in the CSER community, should provide education and guidance on the appropriate applications and limitations of GS and availability of GS resources and referrals to genetic providers.

The potential for complex results requiring extensive preparation has been cited as an anticipated challenge of disclosing results for GS [25]. While initially some of the respondents noted these concerns, they were attenuated with experience. The experiences of the respondents in this study highlight the need to continue to support collaboration across genomic providers to allow for a shorter learning curve and more efficient practice. Continued emphasis on data sharing through databases such as ClinVar [26], a database of suspected pathogenic variants, expansion of population variant databases such as, Exome Aggregation Consortium [27] and Exome Variant Server [28] and methods to match clinicians and researchers working with rare disorders like, GeneMatcher [29] is critical for increasingly efficient and accurate variant interpretation as well as access to clinical course data that can potentially offer providers more information to incorporate into patient education and counseling.

The respondents’ descriptions of the participant’s varied reactions to results are consistent with published reports of patient and participant experiences [13, 30–32]. The experience of receiving diagnostic results is multifaceted and shaped by the classification of results, participant expectations, health status and prior experience with genetics. Additionally some of the patient needs identified by the respondents are similar to those reported by patients which include a desire for a thorough explanation, guidance regarding medical management and resources, empathy and ongoing, open communication with the provider [30]. Respondents reported difficulties returning rare diagnostic results when there is limited or no information about prognosis; this is mirrored in patient reports of frustration of lack of information and limitations of results to guide medical management [31].

The challenge of modulating the participants’ expectations, first identified in studies of consenting for GS [11, 33], continued to be cited as a significant challenge in the disclosure sessions. Unrealistic expectations may be amplified in genomics due to the breadth and inherent uncertainty of test results and can cause feelings of disappointment and sometimes anger or mistrust. The media portrayal of the comprehensiveness of GS technology, as well as the provider’s presentation of the test, may foster these unrealistic expectations. Respondents frequently reflected on how greater familiarity with the types and frequency of results and experience in the disclosure sessions helped them to better calibrate participant expectations during consent sessions. Data from large cohort studies of GS recently have defined the diagnostic rate for various indications [34–41] and the potential for secondary findings [42], allowing providers to help patients develop realistic expectations of sequencing results by communicating more precise diagnostic yields as well as the limitations of the test.

The increased rate of identification of VUS is a unique characteristic of GS. Respondents reported that, similar to negative results, some participants with these types of results were disappointed or frustrated to not have a clear answer but did not report these feelings negatively affected their relationship with the patient. In fact, some respondents felt conversations about potential reclassification of VUS helped to build a relationship of trust by demonstrating that the patient-provider relationship will continue over time as more is learned about genetic variants and as technology improves. These feelings have also been reported by patients [30]. Interestingly, multiple respondents reflected on decreasing the emphasis on VUS results as they felt that too much explanation led to over interpretation of the significance. There was a need to calibrate the participants’ level of concern over the uncertainly of a VUS while balancing the concept that, at this time, no medical action is indicated but there is the potential for this to change.

Finally, the unique potential for reanalysis and reinterpretation of negative and VUS results from GS has been discussed [16, 33]. While few respondents had experience returning new results from reanalysis, the possibility of reanalysis was discussed in some of the disclosures sessions and results letters. Several respondents reflected that the concept of reclassification was difficult to understand and added to the difficulty of managing uncertainty but was also affected by the participant’s tolerance for ambiguity. These challenges were amplified by respondent’s own uncertainty around reclassification. In general, re-analysis was not reported to be a significant part of most sessions and this may have been in part due to the timing of interviews in the course of the study, the research nature of the results and finite length of the study. Future studies that specifically study the experience of re-analysis are needed to further understand how this affects the patient experience and provider-patient relationship.

The potential of secondary results to negatively affect patients and participants by revealing unwanted, overwhelming or uncertain information and the possibility of misinterpretation and inappropriate medical care was initially very concerning to the genomic community [43–47]. Despite earlier concerns, the most frequently reported response was disappointment when no secondary results were identified. Respondents did not report any significant negative experiences or frequent over-interpretation of the significance of personal health secondary risks. The general lack of adverse impact of learning secondary findings is consistent with early research on participant experiences and suggest that, with appropriate counseling, participants are comfortable with learning these types of results [32]. One respondent did express concern about overwhelming the participant when both diagnostic and secondary results were returned and the potential that participants will not follow up on recommendations. It has been proposed that this could be addressed with a staged disclosure of results but this method and potential obstacles of participant and provider availability and patient compliance has not systematically evaluated [48].

As the growth in use of GS continues, more providers will be using this technology including non-genetic providers. Respondents agreed that there is a need to make genomic education and resources more accessible. The CSER consortium “Guide to Interpreting Genomic Reports” is a comprehensive review of different types of genomic results with links to additional resources and is an important resource for providers new to GS [49] . As genomic medicine diffuses beyond traditional genetics settings, it will be the responsibility of genomic providers to be accessible to non-genetic providers, lead endeavors to educate the non-genomic community and help them to recognize appropriate referrals to a genetic provider.

### Limitations

Several factors may have limited this research. Only a small number of clinicians were interviewed, and respondents were all from CSER consortium projects and may not fully represent the diversity of providers disclosing results from GS or the patient populations being disclosed to but this was not the intention of this study. In particular, the non-genetic providers included in the study did not have formal training in genetics though they had genetics education as part of the study and therefore their experiences may not reflect those of the larger population of non-genetic providers. While we had a modest response rate of 70% and attempted to recruit a variety of provider types from each site (when multiple provider types were involved), those providers who did not participate may have had a different experience. The respondents recall may not fully reflect the experience of results disclosure, particularly the experience of the participants.

Additionally, the patient population of the CSER consortium represented primarily Caucasian and highly educated individuals receiving care at mostly large academic institutions. The experience of this study population may not be representative of more culturally and ethnically diverse populations or underserved patient populations. Further studies such as All of Us [5] and the Clinical Sequencing Evidence Generating Research Consortium [6] will contribute to the knowledge base of the RoR process across more diverse populations.

## Conclusions

The experiences of returning results from GS, as found in this study, provide insight for other providers and will help to guide the development of best practices for results disclosure. These experiences emphasize the ongoing need to manage patient expectations throughout the process. They also provide important reflections on the unique challenges of disclosing multiple results, including secondary and uncertain results. The evolution of the process as the providers gained experience is particularly important for preparing future providers utilizing GS. There is need to examine the participant experience of results disclosure including understanding and psychological experience, as well as participants’ assessment of clinical and social utility. There is also a need to more fully explore the experience of returning results following re-analysis and re-interpretation. Finally, there is a continued need to extend these observations to include diverse, underrepresented and underserved populations in order to determine how to best implement GS into the care of all patient populations.

## Additional file

Additional file 1: Pre-interview email and interview script. (DOCX 99 kb)

## Abbreviations

CSER: Clinical sequencing exploratory research
GC WG: Genetic counseling working group
GC: Genetic counselor
GS: Genomic sequencing
RoR: Return of results
VUS: Variants of uncertain significance

## References

[1] Farwell KD, Shahmirzadi L, El-Khechen D, Powis Z, Chao EC, Davis BT, Baxter RM, Zeng W, Mroske C, Parra MC (2014). Enhanced utility of family-centered diagnostic exome sequencing with inheritance model-based analysis: results from 500 unselected families with undiagnosed genetic conditions. Genet. Med.

[2] Lee H, Deignan JL, Dorrani N, Strom SP, Kantarci S, Quintero-Rivera F, Das K, Toy T, Harry B, Yourshaw M (2014). Clinical exome sequencing for genetic identification of rare Mendelian disorders. JAMA.

[3] Yang Y, Muzny DM, Xia F, Niu Z, Person R, Ding Y, Ward P, Braxton A, Wang M, Buhay C (2014). Molecular findings among patients referred for clinical whole-exome sequencing. JAMA.

[4] Iglesias A, Anyane-Yeboa K, Wynn J, Wilson A, Truitt Cho M, Guzman E, Sisson R, Egan C, Chung WK (2014). The usefulness of whole-exome sequencing in routine clinical practice. Genet. Med.

[5] All of US. 2017. https://allofus.nih.gov/.

[6] Clinical Sequencing Exploratory Research Consortium. 2017. https://cser-consortium.org/.

[7] Green RC, Goddard KA, Jarvik GP, Amendola LM, Appelbaum PS, Berg JS, Bernhardt BA, Biesecker LG, Biswas S, Blout CL (2016). Clinical sequencing exploratory research consortium: accelerating evidence-based practice of genomic medicine. Am J Hum Genet.

[8] Facio FM, Sapp JC, Linn A, Biesecker LG (2012). Approaches to informed consent for hypothesis-testing and hypothesis-generating clinical genomics research. BMC Med Genet.

[9] Ayuso C, Millan JM, Mancheno M, Dal-Re R (2013). Informed consent for whole-genome sequencing studies in the clinical setting. Proposed recommendations on essential content and process. Eur. J. Hum. Genet.

[10] Tabor HK, Stock J, Brazg T, McMillin MJ, Dent KM, Yu JH, Shendure J, Bamshad MJ (2012). Informed consent for whole genome sequencing: a qualitative analysis of participant expectations and perceptions of risks, benefits, and harms. Am J Med Genet A.

[11] Bernhardt BA, Roche MI, Perry DL, Scollon SR, Tomlinson AN, Skinner D (2015). Experiences with obtaining informed consent for genomic sequencing. Am J Med Genet A.

[12] Appelbaum PS, Parens E, Waldman CR, Klitzman R, Fyer A, Martinez J, Price WN, Chung WK (2014). Models of consent to return of incidental findings in genomic research. Hast Cent Rep.

[13] Amendola LM, Lautenbach D, Scollon S, Bernhardt B, Biswas S, East K, Everett J, Gilmore MJ, Himes P, Raymond VM (2015). Illustrative case studies in the return of exome and genome sequencing results. Per Med.

[14] Biesecker BB, Klein W, Lewis KL, Fisher TC, Wright MF, Biesecker LG, Han PK (2014). How do research participants perceive “uncertainty” in genome sequencing?. Genet. Med.

[15] Tomlinson A, Skinner D, Perry D, Scollon S, Roche M, Bernhardt B. “Not tied up neatly with a bow”: professionals’ challenging cases in informed consent for genomic sequencing. J Genet Couns. 2015:1–11. 10.1007/s10897-015-9842-8 PMC4621265 25911622

[16] Skinner D, Raspberry KA, King M. The nuanced negative: meanings of a negative diagnostic result in clinical exome sequencing. Sociol. Health Illn. 2016;38(8):1303–17. 10.1111/1467-9566.12460 PMC5089912 27538589

[17] NVivo Qualitative Data Analysis [Software]. Sociol. Health Illn. In., Version 11 edn: QSR internationSociol. Health Illn. al Pty ltd; 2016. https://www.qsrinternational.com/nvivo/home.

[18] Braun V, Clarke V (2008). Using thematic analysis in psychology. Qual Res Psychol.

[19] Kalia SS, Adelman K, Bale SJ, Chung WK, Eng C, Evans JP, Herman GE, Hufnagel SB, Klein TE, Korf BR (2017). Recommendations for reporting of secondary findings in clinical exome and genome sequencing, 2016 update (ACMG SF v2.0): a policy statement of the American College of Medical Genetics and Genomics. Genet. Med.

[20] Green RC, Berg JS, Grody WW, Kalia SS, Korf BR, Martin CL, McGuire AL, Nussbaum RL, O'Daniel JM, Ormond KE (2013). ACMG recommendations for reporting of incidental findings in clinical exome and genome sequencing. Genet. Med.

[21] Berg JS, Amendola LM, Eng C, Van Allen E, Gray SW, Wagle N, Rehm HL, DeChene ET, Dulik MC, Hisama FM (2013). Processes and preliminary outputs for identification of actionable genes as incidental findings in genomic sequence data in the clinical sequencing exploratory research consortium. Genet. Med.

[22] Online Mendelian Inheritance in Man®. 2017. https://www.omim.org/.

[23] GeneReviews® 2017. https://www.ncbi.nlm.nih.gov/books/NBK1116/.

[24] Djurdjinovic L, Uhlmann W, Schuette J, Yashar B (2009). A guide to genetic counseling. Second Edition edn. Hoboken.

[25] Biesecker LG (2012). Opportunities and challenges for the integration of massively parallel genomic sequencing into clinical practice: lessons from the ClinSeq project. Genet. Med.

[26] ClinVar. 2017. https://www.ncbi.nlm.nih.gov/clinvar/.

[27] ExAC Browser (Beta)| Exome Aggregate Consortium. 2017. http://exac.broadinstitute.org/].

[28] NHLBI GO Exome Sequencing Project (ESP). 2017. http://evs.gs.washington.edu/EVS/.

[29] Gene Matcher. 2017. https://genematcher.org/.

[30] Rosell AM, Pena LD, Schoch K, Spillmann R, Sullivan J, Hooper SR, Jiang YH, Mathey-Andrews N, Goldstein DB, Shashi V. Not the end of the odyssey: parental perceptions of whole exome sequencing (WES) in pediatric undiagnosed disorders. J Genet Couns. 2017;25(5):1019–31. 10.1007/s10897-016-9933-1 26868367

[31] Krabbenborg L, Vissers LE, Schieving J, Kleefstra T, Kamsteeg EJ, Veltman JA, Willemsen MA, Van der Burg S (2016). Understanding the psychosocial effects of WES test results on parents of children with rare diseases. J Genet Couns.

[32] Lewis KL, Hooker GW, Connors PD, Hyams TC, Wright MF, Caldwell S, Biesecker LG, Biesecker BB (2016). Participant use and communication of findings from exome sequencing: a mixed-methods study. Genet. Med.

[33] Amendola LM, Dautenbach D, Scollon S, Bernhardt B, Biswas S, East K, Everett J, Gilmore MJ, Himes P, Raymond VM (2015). Illustrative case studies in the return of exome and genome sequencing results. Pers. Med.

[34] Posey JE, Rosenfeld JA, James RA, Bainbridge M, Niu Z, Wang X, Dhar S, Wiszniewski W, Akdemir ZH, Gambin T (2016). Molecular diagnostic experience of whole-exome sequencing in adult patients. Genet. Med.

[35] Powis Z, Farwell KD, Alamillo CL, Tang S (2016). Diagnostic exome sequencing for patients with a family history of consanguinity: over 38% of positive results are not autosomal recessive pattern. J Hum Genet.

[36] Kuperberg M, Lev D, Blumkin L, Zerem A, Ginsberg M, Linder I, Carmi N, Kivity S, Lerman-Sagie T, Leshinsky-Silver E (2016). Utility of whole exome sequencing for genetic diagnosis of previously undiagnosed pediatric neurology patients. J Child Neurol.

[37] Seidelmann SB, Smith E, Subrahmanyan L, Dykas D, Abou Ziki MD, Azari B, Hannah-Shmouni F, Jiang Y, Akar JG, Marieb M, et al. Application of whole exome sequencing in the clinical diagnosis and Management of Inherited Cardiovascular Diseases in adults. Circ Cardiovasc Genet. 2017;10(1):1–9. 10.1161/CIRCGENETICS.116.001573 PMC5245580 28087566

[38] Haer-Wigman L, Van Zelst-Stams WA, Pfundt R, van den Born LI, Klaver CC, Verheij JB, Hoyng CB, Breuning MH, Boon CJ, Kievit AJ (2017). Diagnostic exome sequencing in 266 Dutch patients with visual impairment. Eur. J. Hum. Genet.

[39] Rossi M, El-Khechen D, Black MH, Farwell Hagman KD, Tang S, Powis Z (2017). Outcomes of diagnostic exome sequencing in patients with diagnosed or suspected autism Spectrum disorders. Pediatric neurology.

[40] Yang Y, Muzny DM, Reid JG, Bainbridge MN, Willis A, Ward PA, Braxton A, Beuten J, Xia F, Niu Z (2013). Clinical whole-exome sequencing for the diagnosis of mendelian disorders. N Engl J Med.

[41] Parsons DW, Roy A, Yang Y, Wang T, Scollon S, Bergstrom K, Kerstein RA, Gutierrez S, Petersen AK, Bavle A. et al. Diagnostic yield of clinical tumor and germline whole-exome sequencing for children with solid tumors. JAMA Oncol. 2016;2(5):616–24. 10.1001/jamaoncol.2015.5699 PMC5471125 26822237

[42] Gambin T, Jhangiani SN, Below JE, Campbell IM, Wiszniewski W, Muzny DM, Staples J, Morrison AC, Bainbridge MN, Penney S (2015). Secondary findings and carrier test frequencies in a large multiethnic sample. Genome Med.

[43] Clarke AJ: Managing the ethical challenges of next-generation sequencing in genomic medicine. Br Med Bull 2014, 111(1):17–30. 10.1093/bmb/ldu017 25122627

[44] Klitzman R, Appelbaum PS, Fyer A, Martinez J, Buquez B, Wynn J, Waldman CR, Phelan J, Parens E, Chung WK (2013). Researchers’ views on return of incidental genomic research results: qualitative and quantitative findings. Genet. Med.

[45] Klitzman R, Appelbaum PS, Chung W (2013). Return of secondary genomic findings vs patient autonomy: implications for medical care. JAMA.

[46] Biesecker LG, Burke W, Kohane I, Plon SE, Zimmern R (2012). Next-generation sequencing in the clinic: are we ready?. Nat Rev Genet.

[47] Burke W, Antommaria AH, Bennett R, Botkin J, Clayton EW, Henderson GE, Holm IA, Jarvik GP, Khoury MJ, Knoppers BM, et al. Recommendations for returning genomic incidental findings? We need to talk! Genet. Med. 2017;15(11):854–9. 10.1038/gim.2013.113 PMC3832423 23907645

[48] Bowdin S, Gilbert A, Bedoukian E, Carew C, Adam MP, Belmont J, Bernhardt B, Biesecker L, Bjornsson HT, Blitzer M (2016). Recommendations for the integration of genomics into clinical practice. Genet. Med.

[49] Guide to Interpreting Genomic Reports: A Genomics Toolkit. http://www.ashg.org/education/csertoolkit/index.html.

