# Supplementary material for: Clinical providers’ experiences with returning results from genomic sequencing: an interview study
Source: BMC Med Genomics. 2018 May 8;11:45. doi: 10.1186/s12920-018-0360-z (PMC5941324; doi:10.1186/s12920-018-0360-z)
Supplement: Supplementary file 1 — Pre-interview email and interview script. (DOCX 99 kb) [file 12920_2018_360_MOESM1_ESM.docx]

Appendix A

**Pre-interview email**

Thank you for your willingness to complete an interview about your experience of returning results from genomic sequencing. We are very grateful to you for taking the time to do this. We are conducting these interview to learn from genetic counselors with the most experience returning genomic results to develop best practices for counselors and other providers who will increasingly be involved genomic sequencing is used more frequently in practice

To prepare for the interview, we would appreciate you reviewing the questions below and providing a brief answer

1. What is your professional training/ background?
2. How many years have you practiced in this profession?
3. What is your primary role? For example, research, clinical or other?
4. Can you comment briefly on the following characteristics of your study population? Age, indication for testing, range of education levels, languages, gender, ethnicities
5. What types of results do you return as part of your study? For example, do you return only results related to the disease indication, do you return incidental/ secondary findings, do you return variants of uncertain significance?
6. What are the most frequent results that are returned as part of your study?
7. Approximately how many results sessions have you been involved in?
8. Does your study re-classify variants and if so do you return them to the participant?
9. Does
10. Do you also return genomic results to non-research patients/ use W/GES in your clinical practice if so who is your patient population?Appendix B

**Semi-structured interview script**

**Introduction and Purpose of the study.**

- **(BEGIN RECORDING)** As you may recall from the letter provided to you in the introductory email to the study, the purpose of the study is to gain an understanding of the task of returning results for genomic sequencing so as to inform the development of guidelines for the process of return of results for sequencing. By responding to the survey questions you provided consent for a one-time telephone interview as a part of the research study.

**Study Participation**

- Your participation is voluntary. It will involve only this one audiotaped telephone interview which will take about 45 minutes. If at any point during the interview you feel uncomfortable or you need to stop the interview, please let me know. You can also decline answering any question if you wish. We will transcribe this interview, but your name and names of any other people or institutions you mention in the interview will be removed from the transcript. Therefore, no one reading the transcript of this interview will know who you are.

**Confidentiality Statement**

- As a participant in this study you will be assigned a unique ID number. The link between your name and ID number will be kept in a separate database that is accessible only to the key study personnel.

**Questions**

- Do you have any questions?

I will now begin the interview.

**Research results returned**

Thank you for taking the time to complete the questions sent to you by email. It is helpful to have this background information going into the phone interview. Let me briefly review your responses. Please provide me with any corrections if I have incorrect information.

I would like to begin the interview by learning more about the role you play at your institution.

1. Can you describe your role in your site’s CSER project for me?

Probes: variant calling, report writing, lead or participate in the results disclosure session

**Process of the preparation and session**

Next I would like to hear more about the preparation that is done before a results disclosure session.

1. Can you tell me about how you prepare for a results disclosure session?

Probes: researcher meetings, primary literature search, database searches, review of the participant case, writing results reports

1. How much time does the preparation take? Are there factors that affect the amount of prep time?

Probes: Are there certain cases/ results/ situations that make the prep time take longer or shorter.

1. Is your site tracking prep time? If yes, how?

**Disclosure session**

Now I want to talk with you about what happens during the disclosure sessions

1. Can you begin by telling me about who is involved in the sessions?

Probes: Besides you, who else is present for the sessions? What is the role of each person in the process?

1. Can you explain to me the flow/ structure of the session(s)?

Probes: How do you open a session? What decides the flow/ order of the session?

1. Are there separate return of results sessions for different types of results (for example diagnostic versus incidental findings)?
2. Are there non-research related portions of the results sessions?
3. Are there multiple sessions for the same type of results?
4. Do you provide educational materials before the session? If so, what are they?

Probes: Do you find these aid the session?

1. Do you use any educational aids in the session. If yes, please describe.

Probes:Have you used materials that were already developed or have you developed any of your own educational tools?

1. What other educational materials do you think would be helpful?
2. How do you assess participant understanding of the results?

Probes: What strategies work best for you/do you use the most?

1. What have been some of the patient-participant’s reactions to the results?

Probes: What are some of the common reactions? Unexpected reactions? Are reactions different depending on the results?

1. What are some of the common questions participants/families ask during the sessions?
2. Are there any common misperceptions of participants?

1. What are some of the challenges of sessions?

Probes: For you? For the participant? Returning results to healthy vs sick individual? (if applicable) Returning results to children vs adults? (if applicable)

1. How do you address those challenges?
2. What are some of the emotional or social needs participants raise during sessions when results are discussed?
3. How do you typically address those needs?
4. What are some of the differences/ challenges of returning a primary results (a results related to the disease of concern) vs an incidental/ secondary result vs a negative results?
5. What documentation of the results or session is provided to the research participant?

Probes: results report? Results letters?

1. Are the sessions documented in the EMR? If yes, how?
2. How long do the return of results sessions take?

Probes: Are there factors that influence the length of the sessions? Do you do other research activities during the session? For example, does this time include a study survey?

Follow up

Now I would like to ask a few questions regarding the follow-up that occurs after the session

1. Is there a formal process to assess if the participant follows up on recommendations? If yes, can you describe this process.
2. Do you assess the patient’s satisfaction with the study/the materials provided? If yes, how?
3. How often are you contacted by families with questions following the results disclosures? What kinds of questions do they have?
4. What are some of the challenges of the follow-up process?

Clinical and research

(FOR COUNSELORS INVOLVED IN CLINICAL COUNSELING)

You mentioned in your email responses that you (have) practice(d) as a clinical counselor. I want to talk a bit about how these sessions are similar or different to your clinical practice.

1. How are these sessions different/similar to sessions in which results from other types of genetic testing other than WGS/WES are being disclosed?

Probes: for example microarray or single gene testing

1. Do your research participants differ from your clinical patients and if so how does this affect the session?
2. If you are using WGS/WES in clinical practice how are the research results disclosure sessions similar/ different?

Probes: Counseling methods, education methods, assessing patient understanding

Evolution in the process

You have been involved in quite a few disclosure sessions. I would like to ask you a few questions about what you have learned from your experience so far.

(For counselors involved in clinical counseling) This can include clinical or research settings

1. Have you made any changes to how you conduct the results disclosure?
2. Has your experience with disclosing results affected your informed consent sessions? (If yes), how?

1. What lessons have you learned as you have gained more experience? What has been most surprising?

Educational Needs

1. How can we prepare current providers and future providers, genetic or non-genetic, for disclosing genomic results?
2. How much time do you think providers need to prep and disclose genomic results?
3. **For GC’s/geneticist only**: What do you see as the role of non-genetics providers in the return of genome scale sequencing results?

Probes: Should non-genetic providers return WGS/WES results? Any exceptions?

**For non-genetics providers only**: Based on your own experiences in return of results, what is your view on the role of non-genetics providers in the return of WGS/WES sequencing results?

Probes: Was returning genomic sequencing results something you felt comfortable with? Are there exceptions to what you would be more or less comfortable with?

Variant Re-classification Specific only for those who respond YES to re-analysis in the email. Can we switch gears for a moment and talk about variant re-classification? In your email responses you answered that your site does re-classified variants.

1. What is your policy for return of re-classified variants?
2. Are any additional studies performed or requested to aid in variant classification (for example, additional blood samples)?
3. Do you counsel participants about the possibility of re-classification? If yes, how?
4. How many variants has your site re-classified?
5. How were they re-classified? For example, variant of unknown significance to pathogenic variant
6. Have you returned re-classified variants to patients?
7. If yes, how did the re-classification change the medical management of the patient?
8. What challenges, if any, did you encounter with this experience?

That concludes my questions. Thank you for taking the time to participate in the study. Do you have any questions before we conclude.
